# Hematologic and Serum Biochemical Characteristics of Belgian Blue Cattle

**DOI:** 10.3390/vetsci11050222

**Published:** 2024-05-16

**Authors:** Hugues Guyot, Damien Legroux, Justine Eppe, Fabrice Bureau, Leah Cannon, Eve Ramery

**Affiliations:** 1 Clinical Department of Production Animals, Fundamental and Applied Research for Animals & Health Research Unit (FARAH), Faculty of Veterinary Medicine, University of Liège, Quartier Vallee 2, Avenue de Cureghem 7A-7D, 4031 Liège, Belgium; justine.eppe@uliege.be; 2 VetAgro Sup Lab Center, 69280 Marcy l’Etoile, France; damien.legroux@vetagro-sup.fr; 3 Laboratory of Cellular and Molecular Immunology, GIGA Institute, Liège University, 4000 Liège, Belgium; fabrice.bureau@uliege.be; 4 VetAgro Sup, University of Lyon, Veterinary Campus, UPSP ICE 2021.A104, 69280 Marcy l’Etoile, France; leah.cannon@vetagro-sup.fr

**Keywords:** bovine, reference values, hematology, blood, biochemistry

## Abstract

**Simple Summary:**

Since they have a unique phenotype, with an approximately 20% increase in muscle mass, we hypothesized that Belgian blue (BB) cows may have hematologic and biochemical characteristics that are significantly different from those of other cattle breeds. However, most available reference intervals in cattle are derived from dairy cows, mainly Holstein Friesian (HF) cows. Therefore, the aim of our study was to measure and compare biochemical and hematologic parameters in clinically healthy BB and HF cows. We studied 183 clinically healthy adult BB and HF cows. Our results confirmed that BB and HF represent different populations from a laboratory perspective. Therefore, we propose the first breed-specific reference intervals for BB, which are essential to improve the health management of this breed.

**Abstract:**

Belgian blue (BB) cattle have an 11-bp deletion in myostatin that causes skeletal muscle hyperplasia and increased muscle mass, leading to a ‘double-muscled’ phenotype. Preliminary data suggest that this phenotype may be associated with breed-specific hematologic and biochemical values. Therefore, in this study, we sought to compare hematologic and serum biochemical parameters in healthy BB and Holstein Friesian (HF) cows and to propose breed-specific reference intervals for BB cows. Hematologic parameters, total protein, creatinine, creatine kinase (CK) and aspartate transaminase (AST) activities, albumin, and globulins were measured in 183 clinically healthy adult BB and HF cows. There were significant differences between BB and HF cows in 17 of 27 measured parameters. BB cows had significantly higher creatinine concentration and CK and AST activities (*p* < 0.001). RBCs, hemoglobin, hematocrit (*p* < 0.001), MCV and lymphocytes (*p* < 0.05) were also significantly higher in BB cows compared with HF cows. The average N/L ratio was greater than 1 in both breeds. These results suggest that BB and HF cows have significantly different clinically relevant hematologic and serum biochemical values, and, therefore, breed-specific reference intervals should be used.

## 1. Introduction

Belgian blue (BB) beef cattle have a unique ‘double-muscled’ phenotype, which is a heritable condition resulting from an 11-bp deletion in the coding sequence for myostatin [1,2]. This mutation causes myocyte hyperplasia, leading to an approximately 20% increase in muscle mass, and also causes multiple other phenotypic changes, including decreased size of the heart, digestive tract, liver, lung, and kidneys [3], decreased stress resistance, and increased insulin resistance [1]. While the double-muscled phenotype has been well characterized, it is unclear whether this phenotype is associated with breed-specific hematologic and biochemical values. Hematologic and biochemical reference intervals may be affected by several factors, including breed, age, and sex [4,5,6,7]. Breed-related differences have been demonstrated in dogs [5,6] particularly in sighthounds [8,9], which are the ‘hypermuscled’ canine counterpart of BB cattle, illustrating the need for breed-specific reference intervals. Several publications have proposed ruminant hematologic and biochemical reference intervals, but most of them are derived from dairy cattle, predominantly from HF cows [10,11,12,13], and none is specific for BB cattle. Therefore, these HF reference intervals may be misleading when assessing BB cows’ health. Furthermore, population data may vary in different geographic locations and, to the best of the authors’ knowledge, there are no reference intervals available for Belgian HF.

The purpose of this study was, therefore, to compare hematologic and biochemical data from healthy BB to those from healthy HF cows and to propose breed-specific reference intervals.

## 2. Materials and Methods

### 2.1. Study Population

This study included 183 adult cows (84 HF dairy cows and 99 BB beef cows).

Criteria for animal selection and exclusion before blood sampling followed the American Society for Veterinary Clinical Pathology (ASVCP) guidelines [14]. The cows came from 20 different herds (10 farms for each breed), all of which were located in Belgium (Wallonia), within 150 km of Liege. All cows were enrolled in a health maintenance program managed by the Ambulatory Clinic of the University of Liege (Clinical Department of Production Animals, Faculty of Veterinary Medicine, University of Liege). They came from herds free from tuberculosis, brucellosis, bovine leukosis, bovine viral diarrhea, and infectious bovine rhinotracheitis. All cows had calved at least once.

### 2.2. Inclusion Criteria

Clinical examinations were performed onsite. Each cow was determined to be clinically healthy based on history regarding immediate illness and prophylaxis; clinical examination results within normal limits [15]; and a negative glutaraldehyde test (coagulation > 15 min, Bovi-ƴ test, ‘masked for review’), indicating a lack of chronic inflammation [16]. No drug had been administered to any of the cows for at least one month prior to blood collection.

### 2.3. Exclusion Criteria

Cows were excluded from the study if they had any known disease, if they were receiving medication at the time of examination, or if the inclusion criteria were not met. Blood samples were excluded if there were clots detected in the EDTA tube during processing, hemolysis detected on gross evaluation, or lipemia or icterus in the serum sample, or if samples were not processed within 8 h of collection.

### 2.4. Sample Collection

Blood was collected from the coccygian vein (Vacutainer©; 16G needle, BD Benelux, Erembodegem, Belgium) into dry (4 mL) and K3-EDTA (4 mL) sample tubes (VACUETTE, Greiner Bio-one, Hannover, Germany), in that order. Throughout the entire protocol, the samples were collected and handled by the same person and according to the same procedure in order to avoid interference.

### 2.5. Preanalytical Handling

Blood samples were kept at room temperature and processed within 8 h of collection. The K3-EDTA sample was used directly, while the dry tube was centrifuged (Universal 16 Centrifuge, Hettich, Tuttlingen, Germany) for 10 min at 1800× *g* per minute. No lipemic or hemolytic samples were processed.

### 2.6. Quality Control and Sample Analysis

All samples were analyzed within 8 h of collection. The EDTA-samples were analyzed on a Cell-Dyn 3700 hematology analyzer (Abbott, Chicago, IL, USA). Serum samples were analyzed on the Respons chemistry analyzer (Dyasis Diagnostic Systems, Holzheim, Germany). Serum protein electrophoresis (SPE) was performed using the Hydragel K20 manual gel electrophoresis system (Sebia Benelux, Brussels, Belgium) to measure serum globulins and albumin (g/L).

Three levels (low, normal, and high) of hematologic quality control (QC) (Abbott) and two levels (normal and pathologic) of biochemical quality control (Randox, Mauguio, France) were performed daily. In addition, air-dried May Grunwald Giemsa-stained blood smears were evaluated by a single observer to visually corroborate instrument measurements and assess red blood cell (RBC), platelet (PLT), and white blood cell (WBC) morphology according to the standard operating procedure of the laboratory. The total observed error (TEo) was compared with the total allowable error (TEa) to determine whether assay performance was satisfactory. The methods, units, TEo, and TEa are shown in Table 1.

### 2.7. Statistical Analysis

Data were visually inspected for outliers. ‘Obvious outliers’ were deleted and ‘suspect outliers’ were retained if they were not considered to be aberrant observations, as recommended by the ASVCP guidelines [14]. Normality was assessed using the Anderson–Darling test. To generate reference intervals, data analysis was performed using MS Excel (Microsoft, Redmond, WA, USA) with the macro-instruction set Reference Value Adviser v1.3 (Ecole Nationale Vétérinaire, Toulouse, France) according to published guidelines [17]. Depending on the distribution of each data set, the non-parametric, parametric, or robust method, with or without Box Cox transformation, was applied to calculate the population-based 95% reference intervals (RIs). When the Anderson–Darling test *p* > 0.05, the distribution was considered normal and the parametric method was applied. When *p* < 0.05, Box Cox transformation was performed. When *p* > 0.05 after transformation, the parametric method after transformation was used, and when *p* remained <0.05 after transformation, the non-parametric method was applied.

According to the recommendations of the Clinical Laboratory Standards Institute (CLSI) and the International Federation for Clinical Chemistry (IFCC) [18], a confidence interval (CI) should not exceed 1/5 the width of the reference interval:width of the confidence interval (WCI)/width of the reference interval (WRI) (WCI/WRI).

GraphPad Prism^®^ version v.10.0.1 was used to compare data from BB cows and HF cows. An unpaired *t*-test was used when data from both populations were normally distributed, and a Mann–Whitney test was used when data from one or both populations were not normally distributed.

## 3. Results

### 3.1. Study Population

A total of 183 cows were recruited for this study. Study population characteristics were similar between breeds and are listed in Table 2.

Two BB cows were excluded, one with aberrant SPE results and one with hyperthermia (39.6 °C).

### 3.2. Hematologic Analysis

Fifty-four EDTA samples from BB cows and 11 samples from HF cows were discarded because of clots in the tube. Therefore, a total of 43 EDTA samples from BB cows and 68 samples from HF cows were analyzed. Among these, a further seven samples from HF cows were excluded from platelet analysis because of large aggregates on smear examination.

Upon microscopic examination of blood smears, there were no abnormalities in leukocyte morphology. The automated and manual counts were not substantially different.

Most hematologic parameters were normally distributed, except red blood cell distribution width (RDW), lymphocyte count, and neutrophil/lymphocyte ratio (N/L) in HF cows and WBC count, monocytes, and eosinophils in BB cows. Very few outliers were detected: one for MCV in HF cows and one for MCHC in BB cows. Hematology reference values were established for BB and HF cows (Table 3 and Table 4).

### 3.3. Biochemical Analysis

Most biochemical parameters were not normally distributed. Only creatinine and alpha, beta, and gamma globulins were normally distributed in both breeds. Furthermore, AST, albumin, and A/G ratio were normally distributed in HF cows. Five outliers were detected in the CK measurements: two in HF cows and three in BB cows. Similarly, five outliers were found in AST activity measurements: three in HF cows and two in BB cows. Our proposed biochemistry reference values for BB cows and HF cows are listed in Table 5 and Table 6.

### 3.4. Comparison of Hematologic Parameters in Belgian Blue and Holstein Friesian Populations

**Figure 1:**
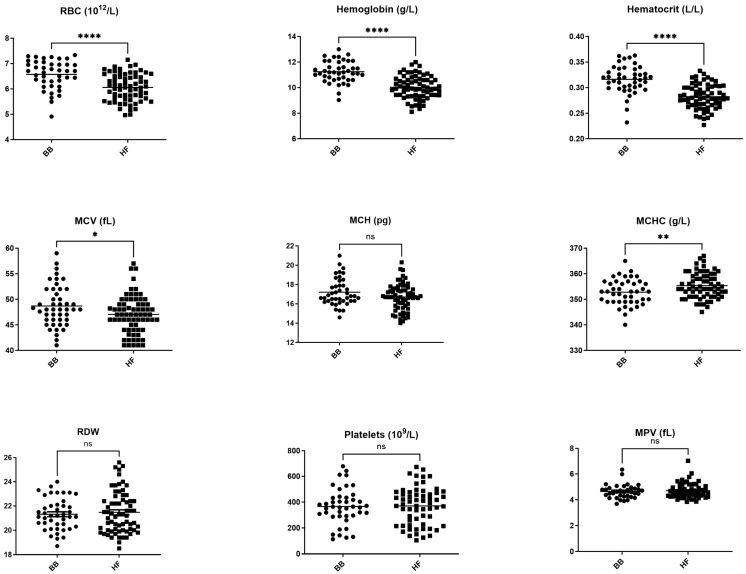
Comparison between RBC and platelet parameters in Belgian blue and Holstein Friesian populations. * *p* < 0.05, ** *p* < 0.01, **** *p* < 0.001, ns: non-significant difference.

**Figure 2:**
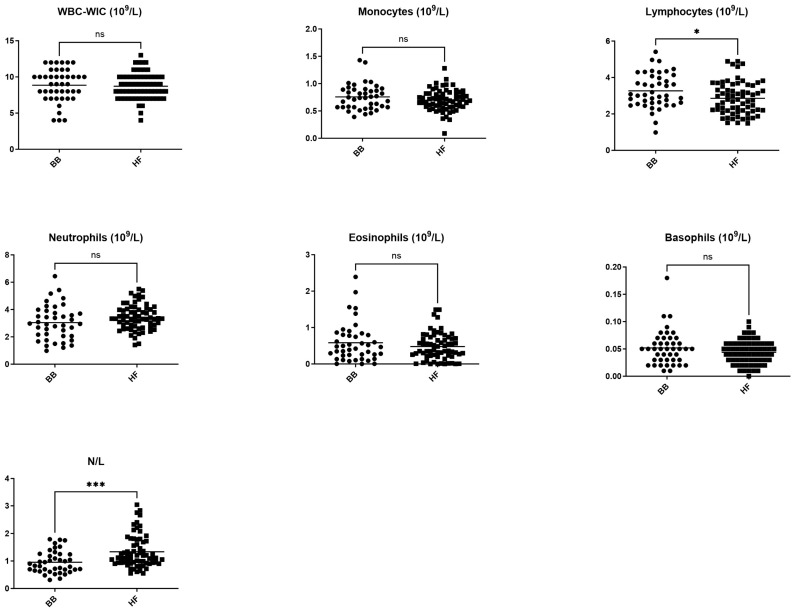
Comparison between leukocyte parameters in Belgian blue and Holstein Friesian populations. * *p* < 0.05, *** *p* < 0.005, ns: non-significant difference.

RBCs, hemoglobin, hematocrit, MCV, and lymphocytes were significantly higher in BB cows compared with HF cows. In contrast, MCHC and N/L ratio were significantly lower in BB cows compared with HF cows (Figure 1 and Figure 2).

### 3.5. Comparison of Biochemistry Parameters in Belgian Blue and Holstein Friesian Populations

**Figure 3:**
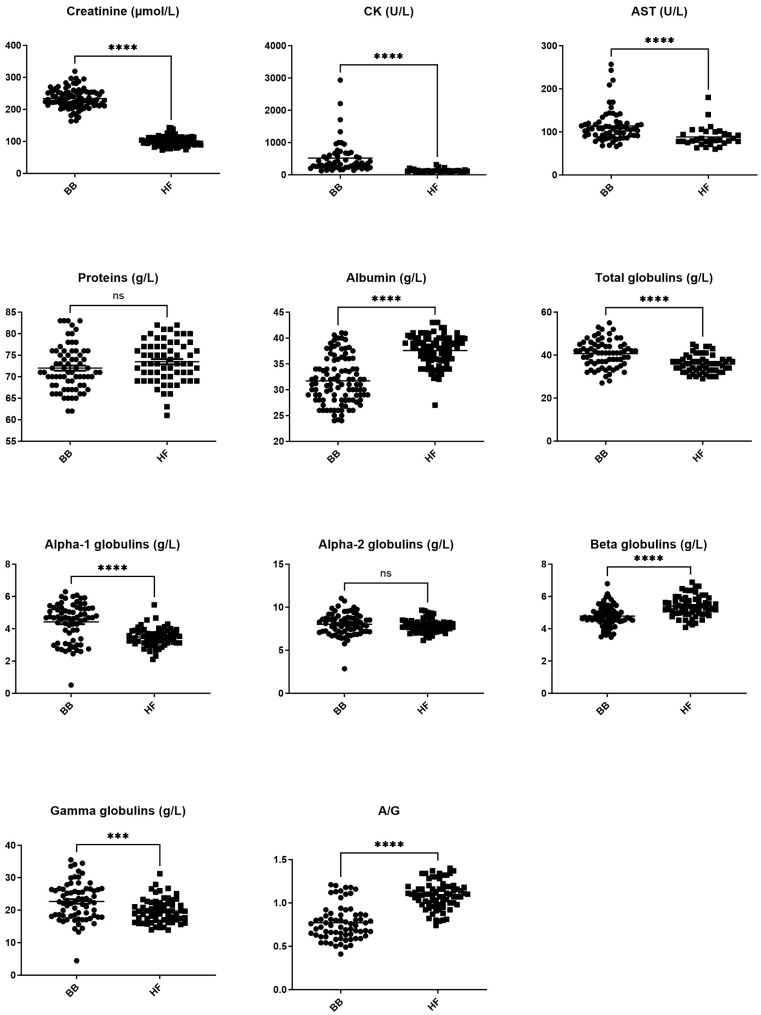
Comparison between biochemistry parameters in Belgian blue and Holstein Friesian populations. *** *p* < 0.005, **** *p* < 0.001, ns: non-significant difference.

Among the biochemistry parameters investigated, only total protein and alpha-2 globulins were not associated with breed. BB cows had significantly higher creatinine, CK activity, AST activity, total globulins, and alpha-1 and gamma-globulins (all *p* < 0.001), while HF cows had significantly higher albumin, beta-globulins, and A/G ratio (all *p* < 0.001, Figure 3).

### 3.6. Comparison of Our Proposed Breed-Specific Reference Intervals with Previously Published Data

**Figure 4:**
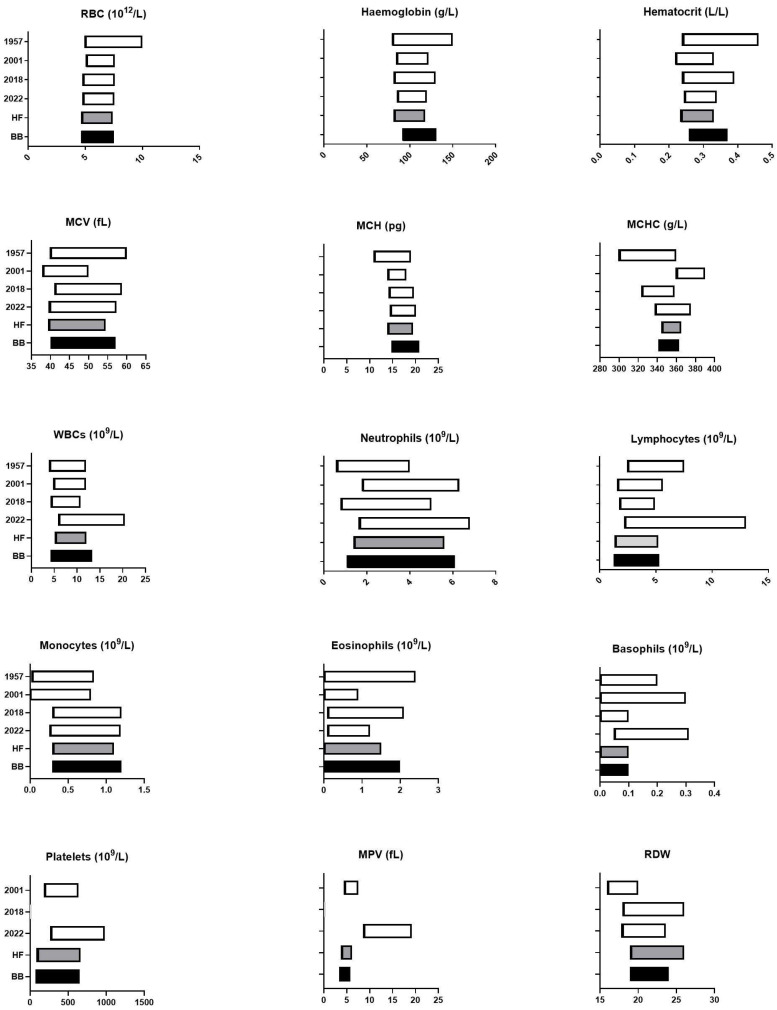
Comparison between our hematology reference intervals for Belgian blue (BB) and Holstein Friesian cattle (HF) and previously published reference intervals obtained from data sets from 1957 and 2001 [9], 2018 [18], and 2022 [19].

**Figure 5:**
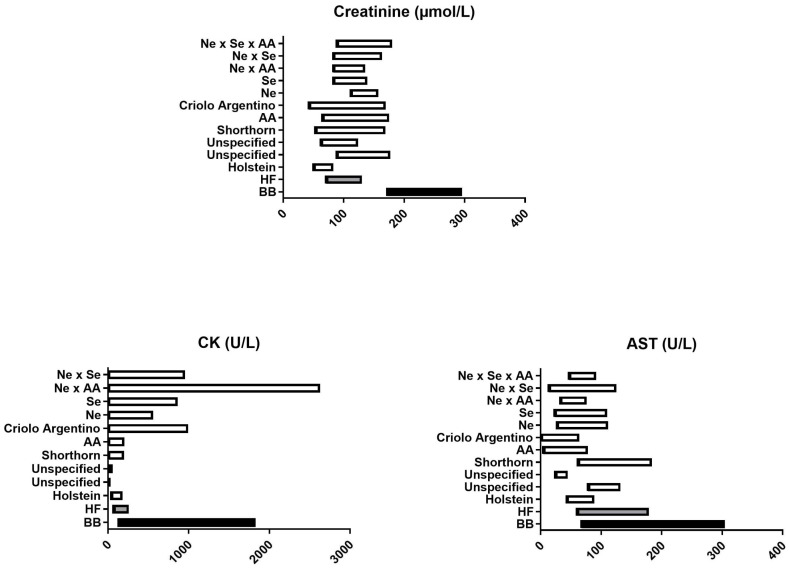
Comparison between our reference intervals directly related to muscle mass in Belgian blue and Holstein Friesian cattle and previously published reference intervals in Holstein cattle [21], unspecified meet breeds cattle [23,24], Shorthorn cattle [4], Aberdeen Angus (AA) and Criolo Argentino [25], Nellore (Ne), Senepol (Se), Ne x AA and Ne x Se crossbreed cattle [26].

**Figure 6:**
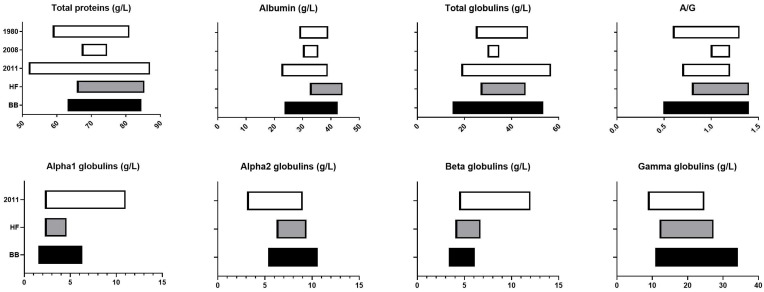
Comparison between our protein-related reference intervals for Belgian blue and Holstein Friesian cattle and previously published reference intervals obtained from data sets from 2011 [11], 2008 [23], and 1980 [24].

In order to position our results within the existing scientific landscape, we then compared our proposed breed-specific reference intervals for BB cows and HF cows with recently published reference intervals (Figure 4, Figure 5 and Figure 6) [4,10,11,19,20,21,22,23,24,25,26].

Our results confirm that BB cows have significantly different hematologic and biochemical values, particularly for analytes directly related to muscle mass, whilst HF results are consistent with recently published data from the literature.

## 4. Discussion

First documented in 1808, Belgian blue is a beef cattle breed from Belgium with a unique phenotype, commonly referred to as ‘double-muscled’. This is a heritable condition resulting from a natural 11-bp deletion in the coding sequence for myostatin protein. The mutation leads to an increase in muscle mass of about 20%, due to muscle hyperplasia [1]. Our data show that the unique phenotype of BB cows results in breed-specific clinical pathologic values. Therefore, breed-specific reference intervals are required to permit accurate assessment of the health of these animals. To the best of the authors’ knowledge, this is the first report of breed-specific hematology and blood biochemistry reference intervals in BB cattle.

When using non-optimized reference intervals, two types of mistakes may occur: either a pathologic change in the analyte is not detected (false-negative result), or a normal level for that specific breed is mistakenly interpreted as a pathologic finding (false-positive result) [5]. Veterinary clinicians rely on reference intervals to decide whether laboratory results indicate pathologic changes in their patients. The use of breed-specific reference intervals for a number of hematologic and biochemical analytes will, therefore, improve patient management for BB cattle.

We found significant differences between BB and HF cows in 17 out of 27 measured parameters (Figure 1, Figure 2 and Figure 3).

Creatinine stands out. Not only did BB cows have significantly higher (*p* < 0.001) creatinine concentrations than HF cows, but also the creatinine reference interval we generated for BB cows does not overlap with our HF reference interval or with published reference intervals from other dairy or beef breeds (Figure 5). These results are not surprising, as creatinine is directly related to muscle mass [4,27,28]. As it is produced at a constant rate [29], serum creatinine concentration is a better indicator of urinary system dysfunction than urea, which varies depending on feed intake, ruminal metabolism, and salivary excretion. Our results highlight the importance of using a BB-specific reference interval for creatinine.

CK and AST activity were also markedly higher in BB cows (*p* < 0.001) compared with HF cows, and this is reflected in the reference intervals. These data are consistent with published data showing wide reference intervals for CK and AST in beef breeds, such as Nellore x Senepol crossbreed cows (Figure 5). AST and CK are both indicators of muscle damage. Unlike creatinine, they are not reported to be routinely elevated simply in the presence of increased muscle mass. The cows in our study showed no clinical signs of muscle disease, such as tremors, muscle contractions, or myoglobinuria. However, AST and CK can also be elevated if animals are restrained, particularly if they are uncooperative. As beef cattle are commonly less managed than dairy cattle, it is possible that they struggled more at the time of sampling. This information was unfortunately not recorded at the time of sampling in our study and is not available in previously published studies [4,21,23,24,25,26]. AST and CK can also be elevated due to hemolysis. Hemolytic interference is unlikely to have influenced our results because we used hemolysis as an exclusion criterion but may be a confounding factor in other studies where the hemolytic status was not reported.

Reference intervals are based on the assumption that the relevant population is relatively homogeneous. Yet, the width of our proposed CK activity reference interval suggests that the variation between individuals is high. Thus, since the width of the reference interval is due to inter-individual variations, the interval is expected to be insensitive to intra-individual variations [30]. However, since 2013, 10 cases of myopathy in adult BB cattle have been diagnosed at the Ruminant Clinic of the University of Liege. These individuals had extremely high CK values, with an average of 120,000 UI/L (min: 2500 UI/L, max: 687,000 UI/L, personal communication), and all would have been detected as abnormal using our reference interval. This supports the use of CK as an indicator of myopathy in BB and other beef cows despite its wide reference intervals. This also highlights the need for appropriate reference intervals for CK and AST in BB cows and other beef breeds. Furthermore, our data suggest that CK measurements should be calculated on individual samples, rather than pooled samples, since a single individual can significantly increase the value of the pool. As CK is specific to muscle damage and rises quickly after muscle injury (within 12–24 h), we suggest repeat assays of the enzyme after 24 h, in conjunction with AST measurement, to diagnose muscle injury in BB cattle [31].

Total proteins were not significantly different (*p* > 0.05) in BB cows compared with HF cows, which is consistent with previously reported data [32,33]. However, there were significantly higher total globulins and lower albumin concentrations in BB cows compared with HF cows (*p* < 0.001), resulting in a significantly lower A/G ratio (*p* < 0.001). Alpha 1 (*p* < 0.001) and gamma-globulins (*p* < 0.005) were higher in BB cows, contributing to the higher total globulins, but beta-globulins were higher in HF cows (*p* < 0.001), despite lower total globulins in this breed. Furthermore, these statistically significant differences (Figure 3) are reflected in the larger width of the reference intervals in BB cows (Figure 5 and Figure 6).

A number of hematology parameters were also significantly different between BB and HF cows. RBC, hemoglobin, and hematocrit were significantly higher in BB cows compared with HF cows (*p* < 0.001). Our results are consistent with previous data showing that beef cattle breeds have higher RBC counts than dairy cattle and that hemoglobin is highly variable between breeds [13]. Similar differences have been reported between hot-blood, cold-blood, and warm-blood horses [34], as well as in sighthounds compared with other dog breeds [8,35]. These breed-specific differences may reflect a higher oxygenation need in animals with an increased muscle mass. This being said, as shown in Figure 4, the RBC reference intervals generated for BB cows and HF cows overlap widely and also overlap with recently published reference intervals [10,32] (with the exception of MCHC and RDW), questioning the clinical utility of using specific reference intervals for these parameters.

We also identified slightly higher MCV and lower MCHC (*p* < 0.05) in BB cows compared with HF cows. These indices help categorize anemia based on cell size and hemoglobin concentration [12]. Our proposed MCHC reference intervals are not significantly different in HF and BB cows, but are narrower than those previously published. As MCHC is very sensitive to interference, particularly hemolysis, this discrepancy may be due to our stringent exclusion of hemolytic samples.

Because the purpose of this study was to address the specificities of BB cows in comparison with HF cows and previously published reference intervals obtained with automated analyzers, we only reported leukocyte differential counts provided by the Cell-Dyn 3700. Therefore, the RIs we generated should be used specifically with the Cell-Dyn 3700 automate. There were no major differences in WBC counts between BB and HF cows, except for lymphocytes, which were slightly higher in BB cows compared with HF cows (*p* < 0.05), resulting in a significantly lower N/L ratio (*p* < 0.005). However, both BB and HF populations had a mean N/L ratio equal to or greater than 1, supporting previous studies suggesting that the inverted N/L ratio should no longer be used to identify inflammation in this species [10,13].

In the ASVCP guidelines [14], the gold standard for establishing reference intervals requires at least 120 samples for each analyte. A small sample size increases the uncertainty of the calculated reference limits. However, reference intervals using less than 120 but more than 40 samples remain acceptable providing that a stringent protocol is used to obtain high-quality samples and robust statistical methods are used to analyze data, both of which were used in this study.

Our findings highlight the need for veterinary laboratories to update bovine hematology and biochemical reference intervals, not only to reflect modern cattle populations, but also to address breed-specific differences. In the present study, our main purpose was to characterize clinical pathology of the BB. However, our results emphasize the need for a larger, more comprehensive study encompassing beef cattle as a whole. More general ‘beef’ reference intervals may be applicable, similar to those used for dairy cattle [35]. We did not investigate differences related to age and physiologic state (gestation, lactation) in this study. However, these data have been previously reported for both dairy and beef cattle [4,7,13]. The variability of hematologic and biochemistry values in bulls also warrants further investigation, as significantly different results are expected in males and females.

## 5. Conclusions

The hematologic and serum biochemistry data of BB cows are significantly different from those of HF cows. Unsurprisingly, this is particularly true for parameters related to muscle mass, including creatinine, CK, and AST. Therefore, the BB-specific reference intervals proposed in this study may help avoid misinterpretation of diagnostic laboratory results in this breed.

## Figures and Tables

**Table 1:** Analytical methods.

Parameter/Analyte	Unit	Method	Analyzer	CV (%)	Bias	Teobs	TEa (%) ASVCP
**HEMATOLOGY**							
Leukocytes	10^9^/L	Impedance	Cell-Dyn 3700, Abbott	5.5	21.8	**32.8**	21.0
Leukocytes	10^9^/L	Optical	Cell-Dyn 3700, Abbott	2.4	3.8	8.6	14.3
Hematocrit	L/L	Calculation	Cell-Dyn 3700, Abbott	2.5	2.3	7.3	10.0
Erythrocytes	10^12^	Impedance	Cell-Dyn 3700, Abbott	1.9	3.7	7.5	10.0
Hemoglobin	g/L	Colorimetric	Cell-Dyn 3700, Abbott	1.4	0.5	**3.3**	1.9
MCV	fL	Impedance	Cell-Dyn 3700, Abbott	1.3	1.1	3.7	7.0
MCH	pg	Calculation	Cell-Dyn 3700, Abbott	1.7	3.9	7.3	NA
MCHC	g/L	Calculation	Cell-Dyn 3700, Abbott	2.0	2.6	6.6	7.0
Platelets	10^9^/L	Impedance	Cell-Dyn 3700, Abbott	3.9	5.2	13.0	25.0
**BIOCHEMISTRY**							
Creatinine	µmol/L	Jaffe rate blanked compensated (−26 µmol/L)	Respons, Dyasis	3.4	6.7	13.5	20.0
CK	U/L	CK-NAC substrate start (DGKC), at 37 °C	Respons, Dyasis	4.7	9.7	19.1	30.0
AST	U/L	Tris buffer with P5P, IFCC/SFBC, at 37 °C	Respons, Dyasis	12.6	8.9	**34.1**	30.0
Proteins	g/L	Biuret reaction, end point	Respons, Dyasis	4.0	4.0	12.0	10.0
Albumin	g/	Serum proteins electrophoresis,	Hydragel protein K20, Sebia cellulose	4.1	3.2	11.4	15.0
Globulins	g/L	Serum proteins electrophoresis	Hydragel protein K20, Sebia cellulose	4.1	4.8	13.0	15.0

TEo: total observed error (bold indicates that the value exceeded the ASVCP-recommended level); TEa: total allowable error; MCH: mean corpuscular hemoglobin; MCHC: mean corpuscular hemoglobin concentration; MCV: mean corpuscular volume; CK: creatine kinase; AST: aspartate aminotransferase; CV (%): coefficient of variation (%); NA: non applicable.

**Table 2:** Characteristics of the study population.

Five HF Cows Were Excluded after Clinical Examination for the Following Reasons: One Was Dehydrated, One Had Severe Microcytosis Indicating Possible Chronic Blood Loss, Two Had Positive Bovi-ƴ Tests, and One Had Severe Leukocytosis.	Stage Preg (M)		Age (Y)		Weight (kg)		BCS (/5)		RR		Rectal Temp	
	HF	BB	HF	BB	HF	BB	HF	BB	HF	BB	HF	BB
Median	4	6	4.5	4	650	650	3	3	32	28	38.5	38.3
Mean	4	7	5	4	646	661	3	3	31	28	38.0	38.0
Minimum	1	1	2.5	2	500	500	1.5	2.5	18	12	37.6	36.7
Maximum	7.5	9	8	7	850	800	4	4	48	56	38.9	39.6

M: months; Y: years; kg: kilograms, stage preg: pregnancy stage in months; RR = respiratory rate (number of respirations per minute); BCS: body condition score using a 5-scale score; Rectal Temp: rectal temperature in degrees Celsius; HF: Holstein Friesian cows (n = 84); BB: Belgian blue cows (n = 99). Age reported in years, weight in kilograms (kg).

**Table 3:** Hematology reference intervals for Belgian blue adult cows.

Parameter	SI Units	n	Mean	SD	Median	Min	Max	*p*-Value ^a^	Distribution ^a^	Method ^a^	LRL of RI	URL of RI	90% CI of LRL	90% CI of URL
PCV	L/L	43	0.32	0.03	0.32	0.23	0.36	0.362	G	P	0.26	0.37	0.25–0.27	0.36–0.38
RBCs	10^12^/L	43	6.5	0.6	6.7	4.9	7.3	0.018–0.634 *	GT	PT	4.7	7.5	ND–5.4	7.3–7.6
Hemoglobin	g/L	43	112	10	112	82	130	0.199	G	P	92	131	88–97	127–136
MCV	fL	43	48.7	4.1	48.3	41.1	59.2	0.339	G	P	40.3	57.1	38.5–42.3	55.3–58.9
MCHC	g/L	42	353	5	353	340	365	0.599	G	P	342	363	340–345	361–365
MCH	pg	43	17.2	1.4	16.8	14.6	21.0	0.02–0.374 *	GT	PT	14.9	20.8	14.6–15.3	19.7–22.1
RDW		43	21.3	1.3	21.2	18.7	24.0	0.401	G	P	19.0	24.0	18.2–19.3	23.4–24.5
WBCs	10^9^/L	43	8.8	2.2	8.8	3.6	12.3	0.295	G	P	4.4	13.3	3.4–5.4	12.2–14.3
Neutrophils	10^9^/L	43	2.9	1.3	2.9	0.5	6.4	0.962	G	P	1.1	6.1	0.9–1.4	5.3–7.0
Lymphocytes	10^9^/L	43	3.3	0.9	3.0	1.0	5.4	0.227	G	P	1.3	5.2	0.9–1.8	4.8–5.6
Monocytes	10^9^/L	42	0.8	0.2	0.7	0.4	1.4	0.1	G	P	0.3	1.2	0.2–0.4	1.1–1.3
Eosinophils	10^9^/L	43	0.6	0.5	0.5	0.0	2.4	0–0.91 *	GT	PT	0.0	2.0	0.0–0.0	1.5–2.6
Basophils	10^9^/L	43	0.1	0.0	0.1	0.0	0.2	0.008–0.393 *	GT	PT	0.0	0.1	0.0–0.0	0.1–0.2
N/L		43	1.0	0.5	0.8	0.2	2.5	0.072	G	P	0.0	2.0	0.0–0.2	1.7–2.1
Platelets	10^9^/L	43	367	139	364	113	679	0.215	G	P	83	650	21–146	586–707
MPV	fL	35	4.6	0.5	4.6	3.7	6.4	0.145	G	P	3.5	5.8	3.3–3.8	5.5–6.0

^a^ Normality was assessed by the Anderson–Darling test. Distribution was considered Gaussian (G) if *p* > 0.05 and the parametric method was applied (P). If *p* < 0.05, Box Cox transformation was performed and transformed data were tested for normality after transformation. If the new *p*-value p* > 0.05, distribution was considered Gaussian after transformation (GT) and the parametric method after transformation (PT) was applied. Otherwise, the non-parametric (NP) method was applied. RI: reference interval; LRL: lower reference limit; URL: upper reference limit; PCV: packed cell volume; RBC: red blood cells; MCV: mean corpuscular volume; MCHC: mean corpuscular hemoglobin concentration; MCH: mean corpuscular hemoglobin; RDW: red blood cell distribution width; WBC: white blood cell; N/L: neutrophil/lymphocyte ratio; MPV: mean platelet volume; 90% CI: 90% confidence interval.

**Table 4:** Hematology reference intervals for Holstein Friesian adult cows.

Parameter	SI Units	n	Mean	SD	Median	Min	Max	*p*-Value ^a^	Distribution ^a^	Method ^a^	LRL of RI	URL of RI	90% CI of LRL	90% CI of URL
PCV	L/L	68	0.28	0.02	0.28	0.23	0.33	0.889	G	P	0.24	0.33	0.23–0.24	0.32–0.34
RBCs	10^12^/L	68	6.1	0.7	6.1	5.0	9.0	0.147	G	P	4.7	7.4	4.5–4.9	7.2–7.7
Hemoglobin	g/L	68	100	9	100	81	12	0.886	G	P	82	118	80–86	115–121
MCV	fL	67	47.0	3.7	46.9	40.5	57.2	0.311	G	P	39.6	54.5	38.3–40.8	53.1–55.7
MCHC	g/L	67	355	5	355	345	367	0.197	G	P	345	365	344–347	363–367
MCH	pg	67	16.7	1.4	16.7	14.0	20.3	0.353	G	P	14.0	19.5	13.5–14.5	19.0–19.9
RDW		67	21.5	1.7	21.3	18.5	25.6	0.008–0.148 *	GT	PT	19	26	18.4–19.1	24.8–27.4
WBCs	10^9^/L	68	8.7	1.7	8.6	4.5	12.5	0.529	G	P	5.3	12.1	4.7–5.8	11.4–12.7
Neutrophils	10^9^/L	68	3.5	1	3.4	1.4	6.7	0.143	G	P	1.4	5.6	1.0–1.8	5.3–6.0
Lymphocytes	10^9^/L	68	2.9	0.9	2.8	1.5	5.7	0.039–0.448 *	GT	PT	1.4	5.2	1.3–1.6	4.6–5.7
Monocytes	10^9^/L	68	0.7	0.2	0.7	0.1	1.3	0.718	G	P	0.3	1.1	0.3–0.4	1.0–1.1
Eosinophils	10^9^/L	68	0.5	0.4	0.4	0	1.5	0.006–0.006	NG	NP	0	1.5	0.0–0.0	1.1–1.5
Basophils	10^9^/L	68	0.0	0.0	0.0	0.0	0.1	0.922	G	P	0.0	0.1	0.0–0.0	0.1–0.1
N/L		68	1.3	0.6	1.1	0.6	3.0	0–0.182 *	GT	PT	0.6	3.0	0.6–0.6	2.5–3.4
Platelets	10^9^/L	61	379	140	392	138	673	0.15	G	P	96	662	49–146	610–712
MPV	fL	61	4.7	0.6	4.6	3.9	7.0	0.006–0.950 *	GT	PT	3.9	6.2	3.8–4.0	5.8–6.7

^a^ Normality was assessed by the Anderson–Darling test. Distribution was considered Gaussian (G) if *p* > 0.05 and the parametric method was applied (P). If *p* < 0.05, Box Cox transformation was performed and transformed data were tested for normality after transformation. If the new *p*-value p* > 0.05, distribution was considered Gaussian after transformation (GT) and the parametric method after transformation (PT) was applied. Otherwise, the non-parametric (NP) method was applied. RI: reference interval; LRL: lower reference limit; URL: upper reference limit; PCV: packed cell volume; RBC: red blood cell; MCV: mean corpuscular volume; MCHC: mean corpuscular hemoglobin concentration; MCH: mean corpuscular hemoglobin; RDW: red blood cell distribution width; WBC: white blood cell; N/L: neutrophil/lymphocyte ratio; MPV: mean platelet volume; 90% CI: 90% confidence interval.

**Table 5:** Biochemistry reference intervals for Belgian blue cattle.

Measurand	SI Units	n	Mean	SD	Median	Min	Max	*p*-Value ^a^	Distri-bution ^a^	Method ^a^	LRL of RI	URL of RI	90% CI of LRL	90% CI of URL
Creatinine	µmol/L	84	234	31	230.5	163	319	0.462	G	P	172	296	162–183	285–305
AST	U/L	66	118	52	107	66	403	0–0	NG	NP	67	304	66–73	212–403
CK	U/L	58	520	503	349	126	2936	0–0.909	GT	PT	131	1830	122–146	1385–2401
Total protein	g/L	70	71.9	5.3	71.0	62.0	89.0	0.012–0.434 *	GT	PT	63.3	84.5	62.2–64.5	81.7–88.0
Albumin	g/L	70	31.7	4.6	31.0	24.2	40.7	0.004–0.052 *	GT	PT	23.8	42.4	22.8–25.0	40.1–44.6
Globulin	g/L	70	32.8	12	32.8	14.6	55.4	0–0	NG	NP	15.2	53.4	14.6–16.4	50.8–55.4
A/G ratio		69	0.8	0.2	0.8	0.5	1.5	0–0.129 *	GT	PT	0.5	1.4	0.4–0.5	1.3–1.6
Alpha-1 globulins	g/L	70	4.4	1.2	4.7	0.5	6.3	0–0.022	NG	NP	1.6	6.3	0.8–2.5	6.1–6.5
Alpha-2 globulins	g/L	70	8	1.3	8.1	2.9	11.0	0.365	G	P	5.4	10.6	5.0–5.9	10.2–11.1
Beta globulins	g/L	70	4.8	0.7	4.7	3.5	6.8	0.479	G	P	3.4	6.1	3.2–3.7	5.9–6.4
Gamma globulins	g/L	70	22.7	5.8	22.6	4.5	35.5	0.218	G	P	11.0	34.3	8.9–12.9	32.2–36.2

^a^ Normality was assessed by the Anderson–Darling test. Distribution was considered Gaussian (G) if *p* > 0.05 and the parametric method was applied (P). If *p* < 0.05, Box Cox transformation was performed and transformed data were tested for normality after transformation. If the new *p*-value p* > 0.05, distribution was considered Gaussian after transformation (GT) and the parametric method after transformation (PT) was applied. Otherwise, the non-parametric (NP) method was applied. AST: aspartate aminotransferase; CK: creatine kinase; A/G ratio: albumin/globulin ratio; 90% CI: 90% confidence interval.

**Table 6:** Biochemistry reference intervals for Holstein Friesian cattle.

Measurand	SI Units	n	Mean	SD	Median	Min	Max	*p*-Value	Distribution ^a^	Method ^a^	LRL of RI	URL of RI ^a^	90% CI of LRL	90% CI of URL
Creatinine	µmol/L	75	101	15	99	73	143	0.152	G	P	71	130	66–76	125–135
AST	U/L	33	92	33	81	60	221	0–0.324 *	GT	PT	60	179	57–64	143–257
CK	U/L	32	134	52	121	71	314	0–0.107 *	GT	PT	70	260	63–79	216–316
Total protein	g/L	62	75.0	5.6	74.0	66.0	86.0	0.012–ND *	GT	NP	66.0	85.4	66.0–67.2	84.9
Albumin	g/L	62	38.5	2.8	38.9	29.3	43.0	0.162	G	P	32.8	44.1	31.8–33.8	43.1–45.0
Globulin	g/L	62	36.5	4.7	36.2	28.7	49.5	0.498	G	P	27.1	45.9	25.5–29.0	44.2–47.5
A/G ratio		62	1.1	0.1	1.1	0.7	1.4	0.529	G	P	0.8	1.4	0.7–0.8	1.3–1.4
Alpha-1 globulins	g/dL	62	3.5	0.6	3.5	2.1	5.5	0.523	G	P	2.3	4.6	2.2–2.5	4.4–4.8
Alpha-2 globulins	g/dL	62	7.8	0.8	7.7	6.1	9.7	0.413	G	P	6.3	9.4	6.0–6.6	9.1–9.7
Beta globulins	g/dL	62	5.4	0.6	5.3	4.1	6.9	0.811	G	P	4.1	6.7	3.9–4.4	6.5–6.9
Gamma globulins	g/dL	62	19.8	3.7	19.6	13.9	31.2	0.111	G	P	12.2	27.3	10.9–13.6	25.9–28.7

^a^ Normality was assessed by the Anderson–Darling test. Distribution was considered Gaussian (G) if *p* > 0.05 and the parametric method was applied (P). If *p* < 0.05, Box Cox transformation was performed and transformed data were tested for normality after transformation. If the new *p*-value p* > 0.05, distribution was considered Gaussian after transformation (GT) and the parametric method after transformation (PT) was applied. Otherwise, the non-parametric (NP) method was applied. AST: aspartate aminotransferase; CK: creatine kinase; A/G ratio: albumin/globulin ratio; 90% CI: 90% confidence interval.

## Data Availability

The raw data supporting the conclusions of this article will be made available by the authors on request.
